# Tuning electronic and magnetic properties of partially hydrogenated graphene by biaxial tensile strain: a computational study

**DOI:** 10.1186/1556-276X-9-491

**Published:** 2014-09-13

**Authors:** Er Hong Song, Ghafar Ali, Sung Ho Yoo, Qing Jiang, Sung Oh Cho

**Affiliations:** 1 Department of Nuclear and Quantum Engineering, Korea Advanced Institute of Science and Technology, Daejeon 305-701, Republic of Korea; 2 Department of Materials Science and Engineering, Jilin University, Changchun 130022, China

**Keywords:** Graphene, Band gap, Magnetism, Strain

## Abstract

Using density functional theory calculations, we have investigated the effects of biaxial tensile strain on the electronic and magnetic properties of partially hydrogenated graphene (PHG) structures. Our study demonstrates that PHG configuration with hexagon vacancies is more energetically favorable than several other types of PHG configurations. In addition, an appropriate biaxial tensile strain can effectively tune the band gap and magnetism of the hydrogenated graphene. The band gap and magnetism of such configurations can be continuously increased when the magnitude of the biaxial tensile strain is increased. This fact that both the band gap and magnetism of partially hydrogenated graphene can be tuned by applying biaxial tensile strain provides a new pathway for the applications of graphene to electronics and photonics.

## Background

Graphene has recently attracted considerable attention owing to its remarkable electronic and structural properties in many emerging application areas such as electronic devices [1-3]. However, graphene exhibits a zero band gap and nonmagnetic behavior, which limits its application in electronics and photonics [4]. Earlier investigations, both theoretically [5-33] and experimentally [34-43], have been made to adjust electronic and magnetic properties of graphene. There are two basic mechanisms cataloged among these schemes, either to disturb the band crossing at Dirac points via breaking the equivalence of the two sublattices of graphene or to transform the carbon hybridization from sp^2^ into sp^3^ via chemical functionalization.

The first mechanism can be achieved by substrate-graphene interaction [5,6,35], applying external electric field [36,37], uniaxial strain [7,8], cutting graphene into nanoribbons [9-11,38] and adsorption of molecules on graphene surface [12-14]. However, the efforts of the abovementioned approaches are limited and can only open a tiny band gap because of the robust π bands of graphene. Another mechanism can be realized via chemical functionalization of graphene, such as H, F, OH, COOH, and O chemisorbed on either one side or both sides of graphene [15-23]. At present, this approach can induce a large band gap opening of graphene: for example, fully hydrogenated graphene has been shown to be a wide band gap semiconductor [16], whereas half-hydrogenated graphene results in an indirect gap and ferromagnetism [26].

Motivated by the above results, we have carried out a systematic investigation to explore the stability and electronic and magnetic properties of partially hydrogenated graphene (PHG) by applying biaxial tensile strain using density functional theory. The calculated results indicate that the configuration with removing H-hexagon is the most energetically favorable in several types of HG configurations, while the appropriate biaxial tensile strain can effectively tune the band gap and magnetism of the partially hydrogenated graphene.

## Methods

All calculations in this study were performed using the spin-polarized first-principle method as implemented in the DMol^3^ code [44]. The generalized gradient approximation (GGA) with the Perdew-Burke-Ernzerhof (PBE) exchange-correlation functional was used [45], in combination with the double numerical plus polarization (DNP). The empirically corrected density functional theory (DFT + D) method within the Grimme scheme was employed in all the calculations to consider the van der Waals forces [46]. All-electron core treatment was adopted, and the real space global cutoff radius was set to be 4.6 Å to achieve high accuracy. We used the smearing techniques with a smearing value of 0.005 Ha.

A hexagonal graphane supercell (7 × 7 graphane unit cell) was established with a lattice parameter of 2.54 Å. The modulus supercell vector in the z direction was set to 15 Å, which led to negligible interactions between the system and their mirror images. For geometric optimization, the Brillouin zone integration was performed with 10 × 10 × 1 k-point sampling, which brings out the convergence tolerance of energy in 1.0 × 10^−5^ hartree, and that of maximum force in 0.002 hartree. The stability of hydrogenated graphene was determined from the formation energy *E*_f_ by the following:

(1) $$E_{f}\left(n\right)\;=E_{\mathrm{total}}\left(n\right)\;+n\mu_{H}-\mu_{\mathrm{graphane}}$$

where *E*_total_(*n*) is the cohesive energy of the system, *n* was the number of H atoms removed from a graphane sheet, and μ_H_ (μ_graphane_) is the chemical potential of the constituent H (graphane) at a given state. Here, we chose the binding energy per atom of H_2_ molecule as μ_H_. And μ_graphane_ was taken as the cohesive energy of a single graphane sheet.

## Results and discussion

According to the previous reports [16,47], two favorable structures of fully hydrogenated graphene (graphene), chair and boat conformations, exist. In the chair configuration, every two adjacent C atoms are hydrogenated from the opposite sides of the graphene sheet. The induced strains compensate each other and thereby the energy of graphane is low. While in the boat conformation, H atoms are alternately bonded to C atoms on both sides in pairs. Due to the repulsion of two neighboring H on the same side, the boat configuration is less stable than the chair one. Therefore, we only consider the chair configuration. For the case of graphane, the obtained lattice parameter, C-C bond length, and C-H bond length of graphane are 2.54, 1.54, and 1.11 Å, respectively, which are in good agreement with previous reported data [25].

**Figure 1:**
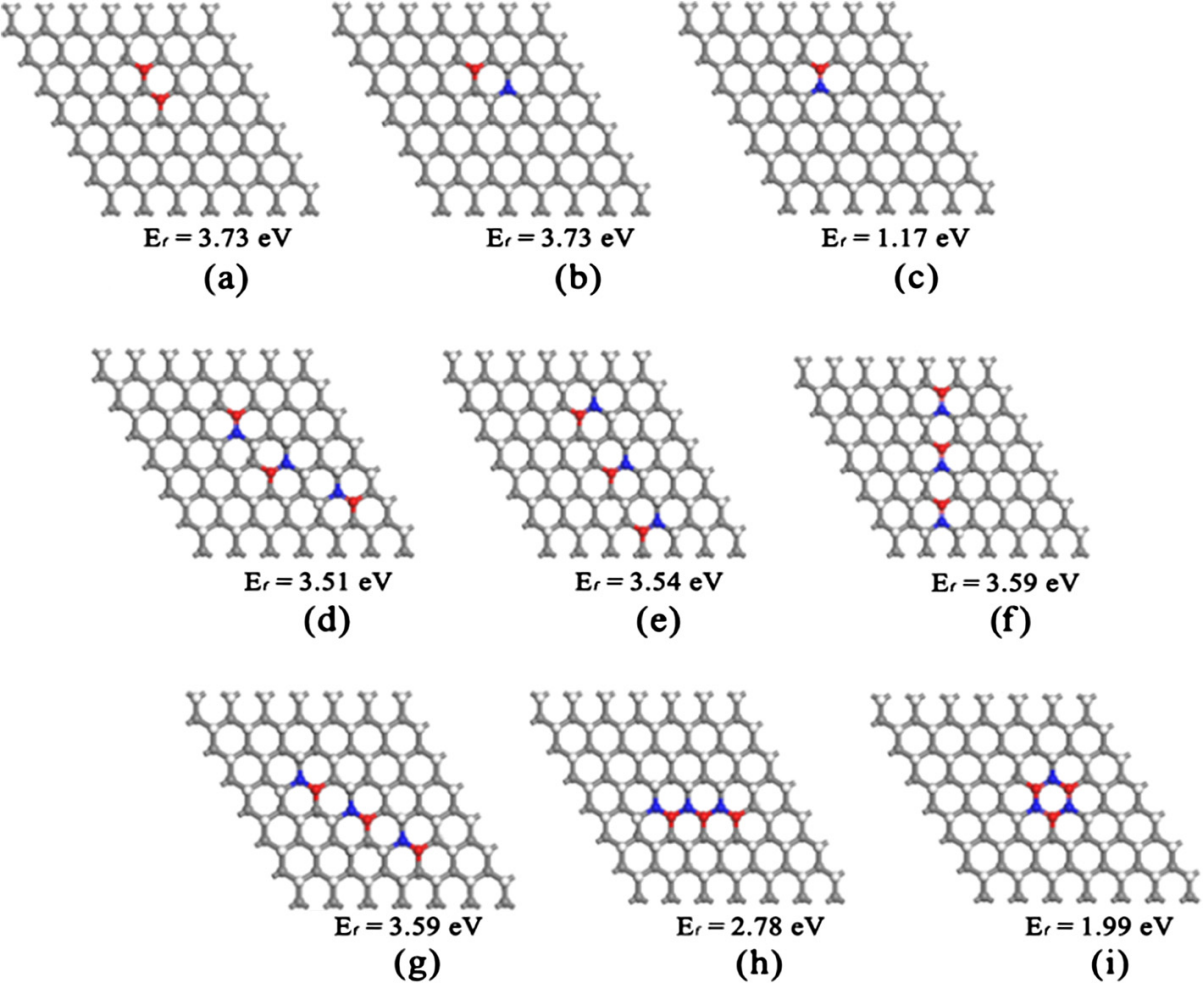
**Optimized geometric structures of partially dehydrogenated graphene with different distribution of H vacancies. (a-c)** individual H atoms are removed from fully HG. **(b-i)** H pairs are removed from fully HG. The gray balls stand for sp^3^ C atoms saturated with H atoms; white balls stand for H atoms, red and blue balls highlight unsaturated sp^2^ C atoms where up and down H atoms are removed, respectively.

As shown in Figure 1a,b,c, we first investigate the relative stability of unpaired (Figure 1a,b) and paired H (Figure 1c) vacancies by calculating the formation energies of the vacancies: H vacancy is isolated in the unpaired vacancy, while two neighboring H vacancies are interconnected in the paired vacancy. *E*_f_ of the H vacancies on graphane can be calculated from the difference between the total energy of graphane and the sum of the total energy of dehydrogenated graphane and those of detached single H atoms. In Equation (1), positive *E*_f_ represents endothermic process of dehydrogenation. The calculations show that *E*_f_ of paired vacancies is always smaller than that of two unpaired vacancies (Figure 1a,b,c). In other words, paired vacancies are more easily formed than unpaired vacancies in graphane. This can be explained by the fact that a single H vacancy creates a dangling bond and raises the total energy due to a local strain [23]. In the condition of paired dehydrogenation, both unsaturated C atoms are turned to sp^2^ hybridized state and form a C = C double bond, through which the two π electrons pair together and the vacancy-induced local strain can be partially released. The removal of radicals by C = C double bond has significant effect on the electronic structures of the dehydrogenated graphane. As shown in Figure 1d,e,f,g,h,i, three adjacent paired vacancies formed a hexagon vacancy and graphane with a hexagon vacancy is more energetically favorable than five separated paired vacancies.

**Figure 2:**
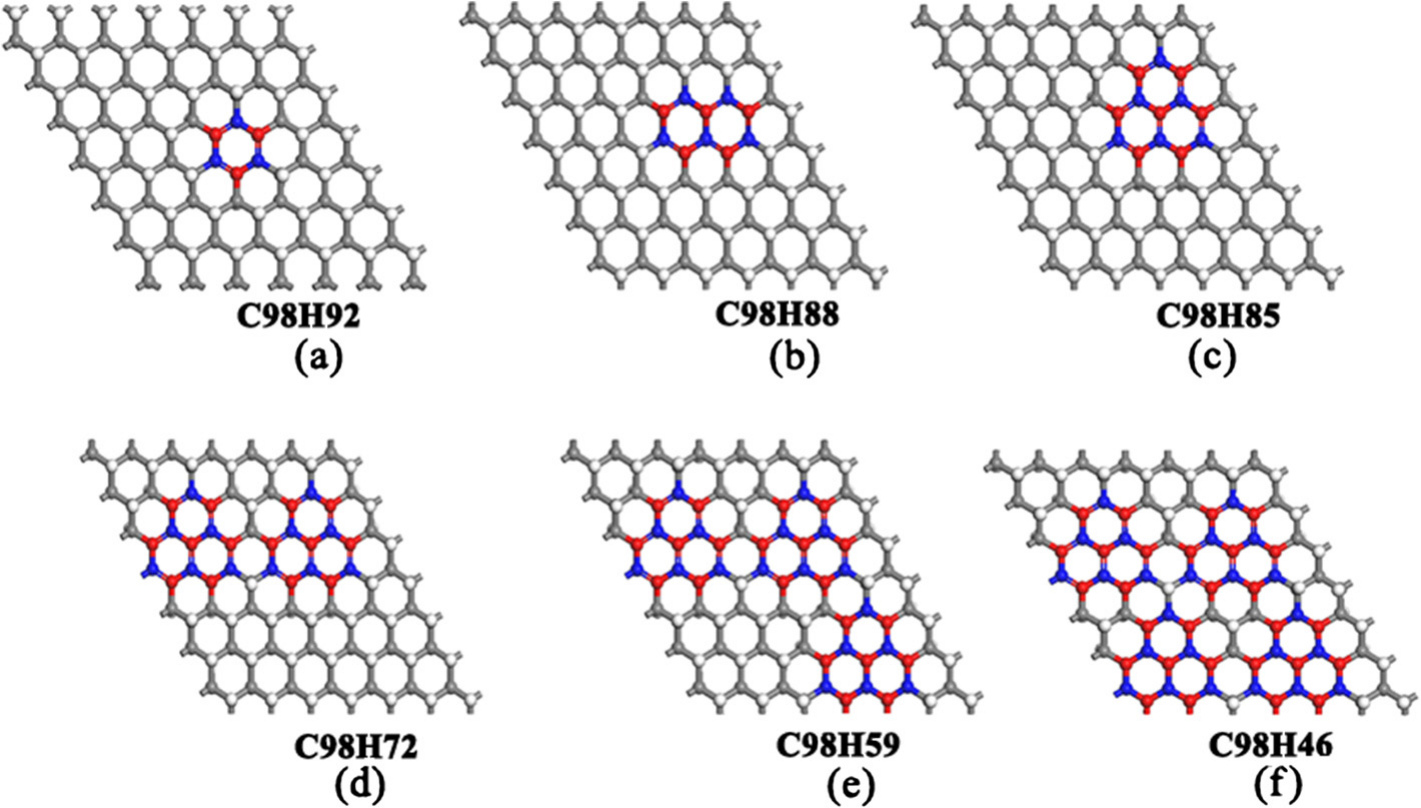
**Optimized geometric structure of dehydrogenated graphane with distribution of H-hexagon vacancies. (a-f)** The structures of C98H92 (1-hexagon vacancy), C98H88 (2-hexagon vacancies), C98H85 (1-triangle vacancy), C98H72 (2-triangle vacancies), C98H59 (3-triangle vacancies), and C98H46 (4-triangle vacancies). The gray balls stand for sp^3^ C atoms saturated with H atoms (white balls), red (blue) balls highlight unsaturated sp^2^ C atoms removing up (down) H atoms

**Figure 3:**
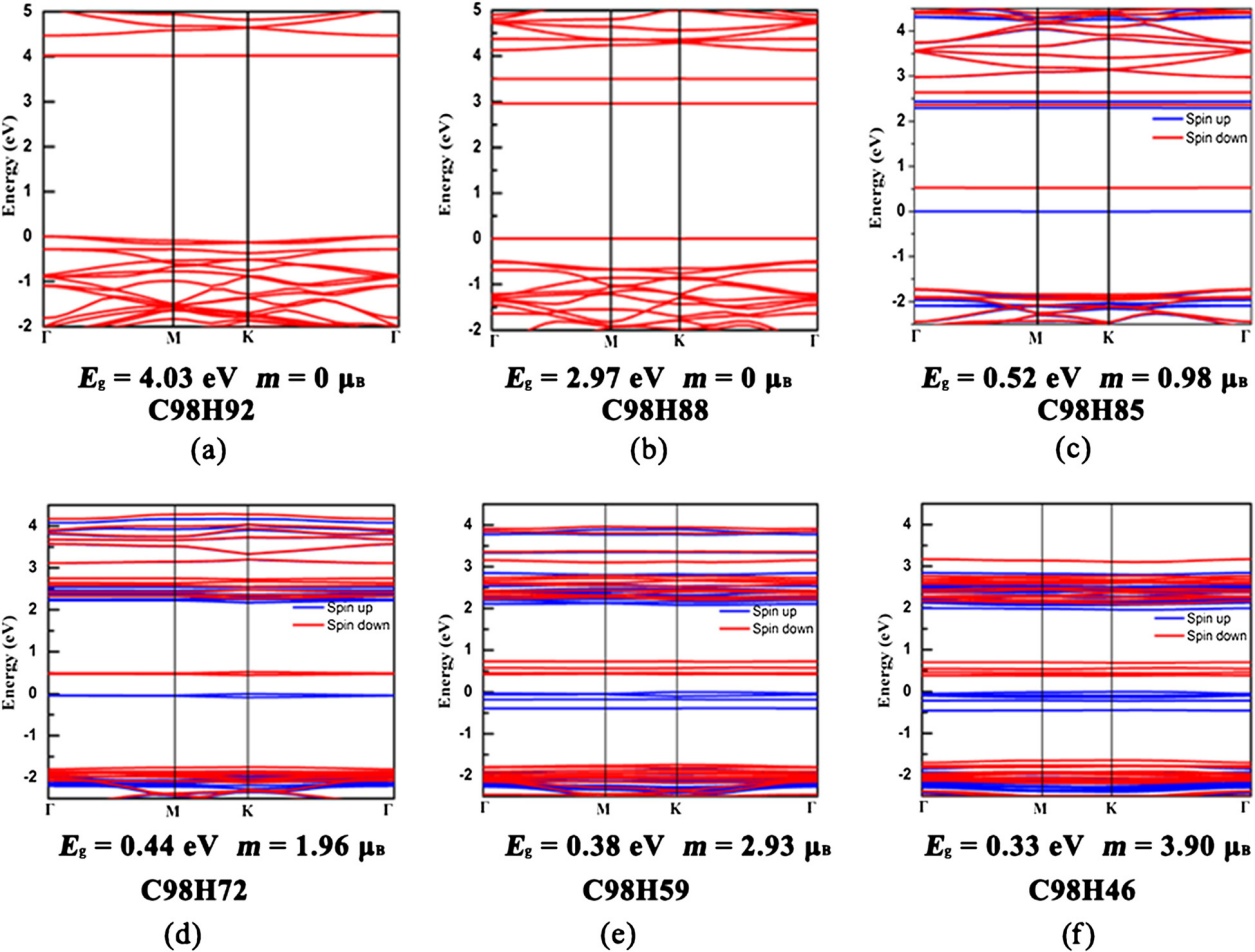
**Electronic structures of dehydrogenated graphane with distribution of H-hexagon vacancies. (a-f)** The band structures of C98H92, C98H88, C98H85, C98H72, C98H59, and C98H46. The blue and red lines represent spin-up and spin-down bands, respectively. And the Fermi level is set to zero.

We further investigated the electronic structures of the partially dehydrogenated graphane with different H vacancies. Previous research works predict that the band gap of perfectly hydrogenated graphene is 3.5 ~ 5.4 eV [16,17,23]. From our calculations, the band gap (*E*_g_) of the perfect graphane is about 4.41 eV. The *E*_g_ opening in graphene can be attributed to the changes of functionalized C atoms from sp^2^ to sp^3^ hybridization. However, after partially dehydrogenation of graphane, the *E*_g_ and the corresponding electronic structures can be changed. According to the unified geometric rule reported by previous works [48,49], the spin state at all zigzag edges with angles of either 0° or 120° between the edges should be ferromagnetic (FM), whereas it should be antiferromagnetic (AFM) if the edges are aligned at angles of 60° and 180° with respect to each other. On the basis of this rule, we calculated the band structures of C98H92 (1-hexagon vacancy, Figure 2a), C98H88 (2-hexagon vacancies, Figure 2b), C98H85 (triangle vacancy formed by 3-hexagon vacancies, Figure 2c), C98H72 (2-triangle vacancies, Figure 2d), C98H59 (3-triangle vacancies, Figure 2e), and C98H46 (4-triangle vacancies, Figure 2f) via selective dehydrogenation. As shown in Figure 3, it is found that the values of *E*_g_ of partially dehydrogenated graphane decrease as the hexagon vacancies increase. Thus, the insulating graphane with a wide band gap becomes a semiconductor via appropriate dehydrogenation. *E*_g_ of PHG which are induced by the quantum confinement effect in PHG with increased vacancies. In other words, the trend of band gap narrowing can be attributed to the number of edge states in the PHG with the hexagon vacancies [22,49-52]. Taking the structure of C98H85 as an example, the *E*_g_ reduces to 0.52 eV and the magnetic moment (*m*) is about 0.98 *μ*_B_ (Figure 3c). When the triangle vacancies of graphane increase to four, the band structure of C98H46 has an indirect band gap with *E*_g_ = 0.33 eV, whereas *m* increases to 3.90 *μ*_B_ (Figure 3f). Meanwhile, the strong σ-bonds are broken between C and H atoms and removal of the nearest neighbor H atoms leads to C = C double bonds, leaving the electrons in the unpaired C atoms localized and unpaired. In order to study the preferred coupling of these moments, we considered the following three magnetic configurations of C98H46: FM coupling, antiferromagnetic (AF) coupling, and nonmagnetic (NM) state, where the calculation is spin unpolarized. From the calculated results, it is found that the total energy of FM state is lower than that of AF and NM states, respectively. Thus, we can deduce that the ground state is FM state.

**Figure 4:**
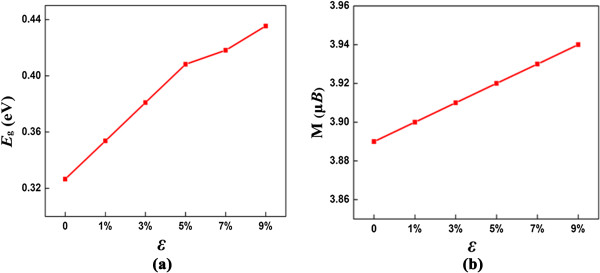
**The band gap and magnetic moment of C98H46 under the biaxial tensile strain. (a)** The band gap of C98H46 as a function of biaxial tensile strain. **(b)** The magnetic moment of C98H46 as a function of biaxial tensile strain.

In the case of the C98H46 model, we apply the external tensile strain to continuously tune the electronic properties of graphene-based materials. Figure 4 shows the influences of tensile strain ϵ on the magnetic and electronic properties of partially hydrogenated graphene (C98H46). It is revealed that the electronic property of C98H46 is rather robust in response to tensile strain. The biaxial strain can be imposed on by changing the lattice parameters of x-y plane and optimized the structures along the z direction. In this work, we considered the tensile strain as it is experimentally more feasible, and the largest tensile strain is chosen to be 9%. Our computations demonstrated that the biaxial strain has a significant impact on the electronic properties of C98H46. With a tensile strain, the *E*_g_ of C98H46 increases monotonically as the increase of strain (Figure 4a), even a 1% strain, can result in a 0.02 eV gap increase. This trend is similar to the case of graphene, as Topsakal et al. demonstrated that the *E*_g_ of graphane monolayer increases with increasing biaxial tensile strain up to 15% [53]. The *E*_g_ of C98H46 system increases to 0.44 eV with tensile strain reaches 9%. Note that when the tensile strain is lower than 9%, C98H46 system is always semiconducting with an indirect band gap. As shown in Figure 4b, we next investigate the influences of tensile ϵ on the magnetic of C98H46 system. Under tensile strain ϵ, C98H46 system always keeps the FM state as in the unstrained case. As ϵ increases to 9%, it is clear that the magnetic moment of C98H46 system slowly increase to 3.94 μ_*B*_*.*

From the above results, we conclude that the electronic and magnetic properties of graphane can be efficiently tuned from insulator to semiconductor (from non-magnetism to ferromagnetism) via selective dehydrogenation, whereas the band gap and magnetism moment of partially hydrogenated graphene can be enhanced by imposing a biaxial tensile strain.

## Conclusions

In summary, DFT calculations with biaxial tensile strain are carried out to investigate the effects of biaxial tensile strain on the electronic and magnetic properties of PHG structures. It is found that the configuration with removing H-hexagon is the most energetically favorable in the several types of HG configurations. In addition, the appropriate biaxial tensile strain can effectively increase the band gap and magnetism of PHG. Overall, tuning both band gap and magnetism of hydrogenated graphene by applying biaxial tensile strain provides a new perspective for wide applications of graphene in electronics and photonics.

## Competing interests

The authors declare that they have no competing interests.

## Authors’ contributions

EHS carried out the design of the simulation and drafted the manuscript. GA, SHY, and QJ assisted in the simulation. SOC supervised the whole study. All authors read and approved the final manuscript.
